# Reduced Field-of-View Diffusion-Weighted Imaging of the Lumbosacral Enlargement: A Pilot *In Vivo* Study of the Healthy Spinal Cord at 3T

**DOI:** 10.1371/journal.pone.0164890

**Published:** 2016-10-14

**Authors:** Marios C. Yiannakas, Francesco Grussu, Polymnia Louka, Ferran Prados, Rebecca S. Samson, Marco Battiston, Daniel R. Altmann, Sebastien Ourselin, David H. Miller, Claudia A. M. Gandini Wheeler-Kingshott

**Affiliations:** 1 NMR Research Unit, Queen Square MS Centre, Department of Neuroinflammation, UCL Institute of Neurology, University College London, London, United Kingdom; 2 Translational Imaging Group, Centre for Medical Image Computing, Medical Physics and Biomedical Engineering, University College London, London, United Kingdom; 3 Department of Medical Statistics, London School of Hygiene and Tropical Medicine, London, United Kingdom; 4 University College London / University College London Hospitals National Institute for Health Research (NIHR) Biomedical Research Centre, London, United Kingdom; 5 Brain MRI 3T Mondino Research Center, C. Mondino National Neurological Institute, Pavia, Italy; 6 Department of Brain and Behavioral Sciences, University of Pavia, Pavia, PV, Italy; Medical University of Graz, AUSTRIA

## Abstract

Diffusion tensor imaging (DTI) has recently started to be adopted into clinical investigations of spinal cord (SC) diseases. However, DTI applications to the lower SC are limited due to a number of technical challenges, related mainly to the even smaller size of the SC structure at this level, its position relative to the receiver coil elements and the effects of motion during data acquisition. Developing methods to overcome these problems would offer new means to gain further insights into microstructural changes of neurological conditions involving the lower SC, and in turn could help explain symptoms such as bladder and sexual dysfunction. In this work, the feasibility of obtaining grey and white matter (GM/WM) DTI indices such as axial/radial/mean diffusivity (AD/RD/MD) and fractional anisotropy (FA) within the lumbosacral enlargement (LSE) was investigated using a reduced field-of-view (rFOV) single-shot echo-planar imaging (ss-EPI) acquisition in 14 healthy participants using a clinical 3T MR system. The scan-rescan reproducibility of the measurements was assessed by calculating the percentage coefficient of variation (%COV). Mean FA was higher in WM compared to GM (0.58 and 0.4 in WM and GM respectively), AD and MD were higher in WM compared to GM (1.66 μm^2^ms^-1^ and 0.94 μm^2^ms^-1^ in WM and 1.2 μm^2^ms^-1^ and 0.82 μm^2^ms^-1^ in GM for AD and MD respectively) and RD was lower in WM compared to GM (0.58 μm^2^ms^-1^ and 0.63 μm^2^ms^-1^ respectively). The scan-rescan %COV was lower than 10% in all cases with the highest values observed for FA and the lowest for MD. This pilot study demonstrates that it is possible to obtain reliable tissue-specific estimation of DTI indices within the LSE using a rFOV ss-EPI acquisition. The DTI acquisition and analysis protocol presented here is clinically feasible and may be used in future investigations of neurological conditions implicating the lower SC.

## Introduction

Diffusion-weighted imaging (DWI) can be used to study the diffusion of water molecules in neural tissue [1]. Among several DWI methods, diffusion tensor imaging (DTI) [2] maps the three-dimensional displacement profile of water molecules due to diffusion, and provides measures that reflect tissue microstructure beyond conventional qualitative magnetic resonance imaging (MRI) assessment. DTI has been used successfully over the years to study neurological conditions affecting the brain [3–5], however the use of DTI to study the spinal cord (SC) has been hampered by a number of technical challenges related mainly to the small cross-sectional size of the structure, susceptibility artifacts introduced by the surrounding vertebral bones and the effect of physiological and involuntary motion during the imaging studies [6, 7].

Technical concerns associated with SC diffusion imaging are related but not limited to the signal-to-noise ratio (SNR), radiofrequency (RF) hardware design, magnetic field inhomogeneities around the SC, cerebrospinal fluid (CSF) pulsation, partial volume effects at the boundary between grey matter (GM), white matter (WM) and CSF and the choice of the acquisition scheme (e.g. type of pulse sequence and readout, number of diffusion directions). Typically, DTI is performed using single-shot echo-planar imaging (ss-EPI) [8]; ss-EPI reduces the sensitivity to subject motion, but causes phase errors to accumulate due to long readout durations. As a consequence, image distortions become evident in the presence of magnetic susceptibility inhomogeneity. This is of particular concern in SC imaging, where tissue susceptibility differences exist in close proximity to the SC, especially due to the vertebral bones. In order to reduce the readout duration and associated off-resonance related image distortions, previous studies included the use of parallel imaging [9, 10], or alternative readout techniques such as interleaved EPI [11], turbo spin echo [12] and line scan imaging [13, 14]. More recently, a number of reduced field-of-view (rFOV) approaches have been proposed for SC imaging [15–21], which offer a significant reduction in the anteroposterior dimension of the imaging volume, thus reducing the readout duration considerably.

DTI with rFOV has been employed successfully to study the upper SC with sufficiently high resolution to distinguish between GM and WM and to allow tissue- or tract-specific estimation of indices such as mean/axial/radial diffusivity (MD/AD/RD) and fractional anisotropy (FA) in the neurologically intact cord [22, 23] and in neurological conditions such as multiple sclerosis (MS) and neuromyelitis optica [24, 25]. However, similar applications in the lower SC are limited, with only a few reports to date [26, 27]. Reliable DTI acquisition and analysis protocols to study the lower SC could provide new insights into the pathophysiology of symptoms such as bladder and sexual dysfunction, which are often associated with neurological conditions such as MS [28, 29], SC injury (SCI) [30, 31] and multiple system atrophy (MSA) [32, 33].

In this pilot study, the feasibility of obtaining tissue-specific (i.e. GM and WM) DTI indices within the lower SC was investigated in a number of healthy volunteers using DTI with rFOV [15, 26] on a clinical 3T MRI system. The DTI acquisition and analysis protocol presented here addresses key methodological considerations such as: i) the positional variation of the lower SC through the use of the lumbosacral enlargement (LSE) as previously suggested [34]; ii) the scan-rescan reproducibility of the DTI indices; iii) the influence of the diffusion encoding protocol on the quality and reproducibility of the DTI indices.

## Materials and Methods

### Study Participants

Fourteen healthy subjects were recruited (6 male and 8 female, mean age 27.3 years; range 21–46 years). The work was approved by the National Hospital for Neurology and Neurosurgery and the Institute of Neurology Joint Research and NRES committee London Bloomsbury (London REC2 Ethics Committee). Written informed consent was obtained from all study participants.

### MR Imaging

A 3T Philips Achieva system with maximum gradient strength 65 mT m^-1^, radiofrequency (RF) dual-transmit technology (Philips Healthcare, Best, Netherlands) and the manufacturer’s product 15-channel SENSE receive-only RF spine coil were used. A conventional T2-weighted image of the lumbar spine in the sagittal plane was first obtained using a turbo spin-echo (TSE) sequence and was used to facilitate prescription of subsequent scans perpendicular to the cord. The imaging parameters for the T2-weighted TSE were: TR = 3575 ms, TE = 100 ms, flip angle α = 90°, FOV = 300 × 180 mm^2^, voxel size = 0.8 × 0.8 × 3 mm^3^, NEX = 2, slices = 15, acquisition time 3:48 min.

Additionally, a 3D slab-selective fast field-echo (3D-FFE) sequence with fat suppression was acquired in the axial-oblique plane (i.e. perpendicular to the longitudinal axis of the cord). The 3D-FFE scan was performed in order to identify the exact location of the LSE, necessary to obtain quantitative measurements of DTI indices. The 3D-FFE slices were prescribed between the T11—L1 level to ensure coverage of the LSE in all cases [34]. The following parameters were used: TR = 22 ms, TE = 4.4 ms, flip angle α = 10°, FOV = 180 × 180 mm^2^, voxel size = 0.5 × 0.5 × 5 mm^3^, NEX = 8, slices = 10, acquisition time 9:51 min.

For estimating the DTI indices within the LSE, a ss-EPI with rFOV was used [15, 26] with cardiac gating and identical slice geometry to the 3D-FFE. Diffusion-weighting was applied along 60 diffusion directions with b = 1000 s/mm^2^, interleaved with 7 b = 0 measurements; Additional parameters for the DTI acquisition were: TR ~ 4000 ms (4 heart beats), TE = 40 ms, flip angle α = 90°, slices = 10; FOV = 64 x 48 mm^2^, voxel size = 1 × 1 × 5 mm^3^, trigger delay for cardiac gating of 150ms. The total acquisition time was ~ 15 min, depending on heart rate.

Motion during the imaging session was minimised with the use of Velcro straps to restrain the torso and with the placement of a large foam wedge beneath the knees, as this increases the level of contact between the lower back and the flat surface of the coil.

### Image analysis

#### LSE identification

Using the 3D-FFE, the three slices (i.e. 15 mm section) covering the widest section of the lumbar cord (i.e. the LSE) were identified, by outlining the cord using the active surface model (ASM) segmentation method available with JIM 6.0 (http://www.xinapse.com), as previously described [34].

#### Motion correction

The diffusion-weighted images were corrected for motion using slice-wise linear registration implemented in FSL (http://www.fmrib.ox.ac.uk/fsl/), with registration transformations estimated among non-DW images, interspersed throughout the diffusion acquisition [23]. Specifically, two degrees of freedom were estimated per slice (i.e. in-plane translation) between each interspersed non-DW image and a mean non-DW image (i.e. the registration target) obtained after a first iteration of the motion correction. Subsequently, each DW image was registered to the target using the transformation of the closest, preceding non-DW image [23].

#### DTI model fitting

DTI was fitted with standard routines in CAMINO (http://cmic.cs.ucl.ac.uk/camino/) and voxel-wise maps of AD, RD, MD and FA were generated. These indices measure: the amount of diffusion along/across the dominant diffusion direction (AD/RD); the mean amount of diffusion (MD); and the anisotropy of the diffusion tensor (a measure of spatial variability of the amount of diffusion) (FA).

#### Image segmentation

Image segmentation was performed with the aim of identifying GM and WM voxels within the three slices of the LSE, in diffusion space. Using the mean b = 0 volume, the three slices corresponding to the LSE, previously identified from the 3D-FFE, were segmented using ASM to obtain the whole cord outline. GM was manually outlined on an image obtained by averaging DW images with good contrast between GM and WM, as previously demonstrated [23, 24]. Binary masks for the GM, WM and whole cord were subsequently created and eroded prior to their application to the DTI maps; WM was defined as the difference between the whole cord and GM masks.

#### Reproducibility assessment

Six out of fourteen volunteers had a repeated scan on a different occasion, with a minimum of 7 days and a maximum of 14 days in between the first and the second visits. For these subjects, the reproducibility of the DTI indices was evaluated within GM, WM and the whole cord by calculating the percentage coefficient of variation (%COV).

#### Influence of the diffusion encoding protocol on the quality and reproducibility of the DTI measurements

MR imaging of the lower SC mostly involves RF coil designs with receiving elements beneath a flat surface. This means that inter-subject variability in positioning the volume of interest (i.e. LSE) with respect to the RF coil surface can influence image quality and therefore the reliability of the DTI measurements, for a predefined diffusion protocol. For instance, high inter-subject variations of SNR due to variable distance between the receiving elements and the volume of interest can cause subject-dependent bias of FA (at moderate b, FA has been reported to increase as SNR decreases [35]). This could ultimately lead to artificial increase of the between-subject variability of this index. On the other hand, for a fixed distance between the receiving elements and the volume of interest, variations of the diffusion protocol in terms of the number of diffusion encoding directions used can affect the estimation of the diffusion properties [36].

In this work, the quality and reproducibility of the DTI indices were evaluated by studying the effect of altering the number of diffusion encoding directions within the same protocol in all subjects. For this purpose, the DTI model was retrospectively fitted to gradually reduced sets of measurements optimally spread over the sphere (60 to 10, with steps of 10) [37], extracted with CAMINO from the original 60-direction scheme.

The quality of the fitted DTI indices was evaluated by visual inspection and by calculating the mean values within each tissue-type (GM, WM, whole cord) in order to quantify the contrast (C) and the contrast-to-noise ratio (CNR) between GM and WM as previously demonstrated [23], using the relations:

$$C=\frac{\left|SI_{WM}-SI_{GM}\right|}{\frac{1}{2}(SI_{WM}+SI_{GM})}$$

and

$$CNR=\frac{\left|SI_{WM}-SI_{GM}\right|}{\sqrt{SD_{WM}{}^{2}+SD_{GM}{}^{2}}}$$

where, SI_WM_, SI_GM_, SD_WM_ and SD_GM_ respectively indicate the mean value of each one of the DTI indices within the WM and GM masks, and the corresponding standard deviation values. C and CNR were calculated in all subjects but without inclusion of repeated scans, which were performed as part of the reproducibility assessment.

Reproducibility (%COV) as a function of the number of diffusion encoding directions was also evaluated in order to establish an adequate number of diffusion-weighted measurements needed to obtain reliable tissue-specific estimation of DTI indices for the population under investigation. DTI indices were assessed in 6 study participants who had repeated scanning, and for whom the cord/coil distance was within the lower and upper bounds previously identified in a larger population (S1 Appendix).

#### Statistical analysis

To obtain the %COV, linear mixed null models were fitted for each measure and for each diffusion encoding scheme, with subject random effect; the %COV was then calculated using the estimated within subject standard deviations and means.

To examine the variation in means of the DTI indices within each tissue-type across the number of diffusion encoding schemes, linear mixed models were fitted over the various diffusion encoding schemes with diffusion encoding scheme as fixed effect; a joint wald test was then used to test the null hypothesis of no variation across the schemes. There was no evidence of departure from model normality assumptions. These models were fitted in Stata 14.1 (Stata Corporation, College Station, Texas, USA).

Possible differences in DTI indices between GM and WM were assessed using paired t-tests, after checking the normality of the paired differences. Statistical significance was accepted at *p*<0.05.

## Results

**Fig 1:**
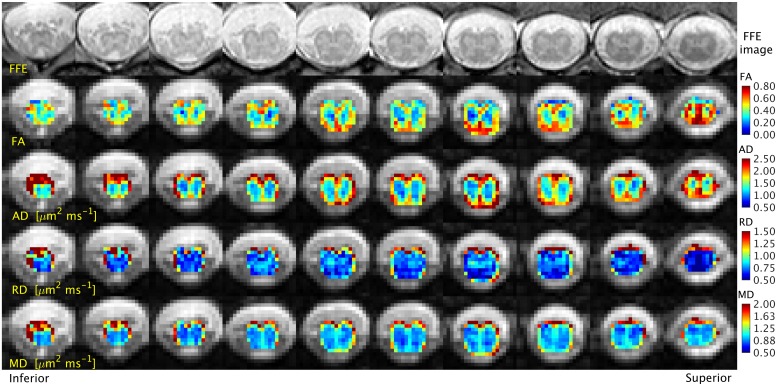
High-resolution images acquired through the T11-L1 level with the 3D fast field-echo (3D-FFE) sequence and the corresponding maps of fractional anisotropy (FA), axial diffusivity (AD), radial diffusivity (RD) and mean diffusivity (MD). The rightmost column corresponds to the T11 level, with contiguous columns moving inferiorly towards L1 (leftmost).

All the study participants tolerated the imaging session well without the need to discard any data because of motion related artifacts. The number of slices obtained in the present study was sufficiently high to ensure coverage of the LSE in all cases, although it was possible that, in some cases (e.g. small-framed individuals), the same number of slices may have covered a section beyond the minimum required, in which case the extra slices were simply discarded. An example of the high-resolution structural images acquired through the T11-L1 level in a single healthy subject and the corresponding quantitative DTI parameter maps are shown in Fig 1.

**Fig 2:**
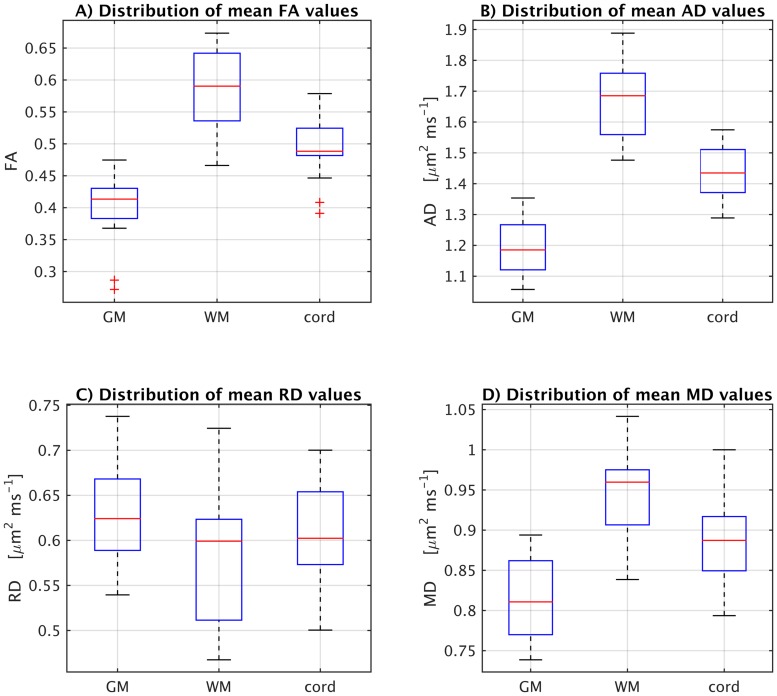
Box plots displaying the distribution within each tissue-type of fractional anisotropy (FA), axial diffusivity (AD), radial diffusivity (RD) and mean diffusivity (MD) values in all study participants.

GM, WM and whole cord values (mean ± SD) of AD, RD, MD and FA in 14 healthy subjects are shown in Table 1. From Table 1 it can be seen that FA is higher in WM compared to GM (mean across 14 participants of 0.58 and 0.40 in WM and GM respectively, p<0.001), AD is higher in WM compared to GM (1.66 μm^2^ms^-1^ and 1.20 μm^2^ms^-1^ respectively, p<0.001), MD is higher in WM compared to GM (0.94 μm^2^ms^-1^ and 0.82 μm^2^ms^-1^ respectively, p<0.001) and RD is lower in WM compared to GM (0.58 μm^2^ms^-1^ and 0.63 μm^2^ms^-1^ respectively, p = 0.007). Separate box plots displaying the distribution of the DTI indices in all study participants are shown in Fig 2.

**Table 1:** DTI indices obtained within the lumbosacral enlargement (LSE); mean (SD) values in grey matter (GM), white matter (WM) and the whole cord in 14 healthy volunteers.

DTI indices	GM	WM	Whole cord	p-value*
FA	0.40 (0.06)	0.58 (0.07)	0.49 (0.05)	p<0.001
AD [μm^2^ ms^-1^]	1.20 (0.87)	1.66 (0.13)	1.43 (0.83)	p<0.001
RD [μm^2^ ms^-1^]	0.63 (0.53)	0.58 (0.22)	0.60 (0.58)	p = 0.007
MD [μm^2^ ms^-1^]	0.82 (0.50)	0.94 (0.54)	0.88 (0.49)	p<0.001

* p-values correspond to differences between GM and WM and were assessed using paired t-tests.

The results from the reproducibility assessment (%COV) using the original acquisition protocol, with diffusion-weighting applied along 60 directions, are reported in Table 2. The %COV was found to be lower than 10% in all cases, with the highest values observed for FA and the lowest for MD.

**Table 2:** The percentage coefficient of variation (%COV) of the DTI indices obtained with the original protocol, with diffusion-weighting applied in 60 directions, is reported separately for grey matter (GM), white matter (WM) and whole cord.

%COV	GM	WM	Whole cord
FA	9.1	6.0	6.7
AD	7.1	5.4	6.3
RD	4.3	8.3	6.4
MD	4.4	5.0	5.0

**Fig 3:**
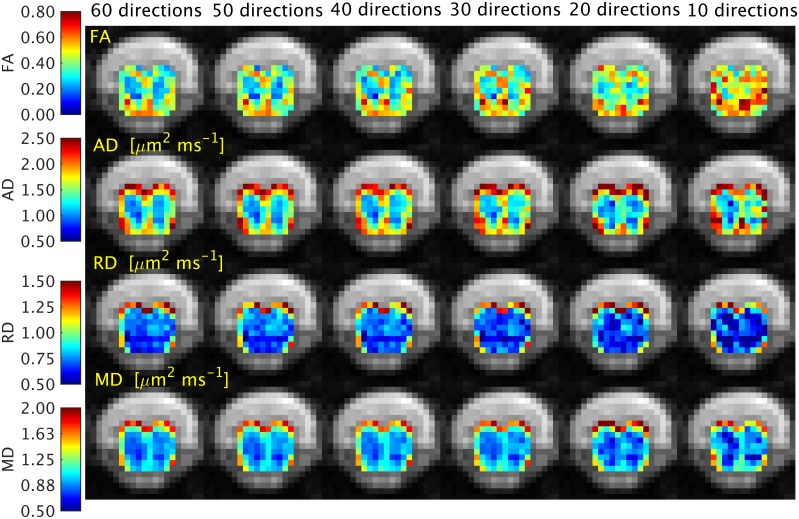
The effect of fitting the DTI model using a different number of diffusion directions on the quality of the DTI parameter maps is demonstrated here in a single study participant.

**Fig 4:**
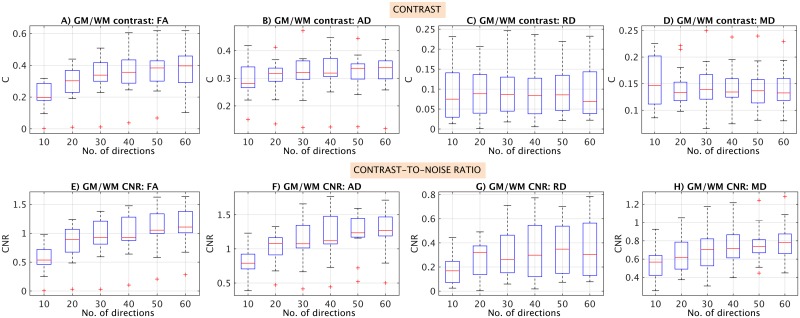
Box plots displaying the distribution of contrast (C) and contrast-to-noise ratio (CNR) values between grey matter (GM) and white matter (WM) for all DTI indices and in all subjects, as a function of the diffusion encoding scheme.

The effect of fitting the DTI model with gradually reduced sets of measurements on the quality of the DTI parameter maps is shown in Fig 3. It is apparent from the figure that the quality of the indices degrades as the number of diffusion directions decreases. The distribution of C and CNR values between GM and WM for all DTI indices and in all subjects as a function of the diffusion encoding scheme are shown in Fig 4. The figure shows that, both for C and CNR, there is a trend towards increasing values when a higher number of diffusion encoding direction is used. This effect appears to be evident across all the DTI indices, with more pronounced improvements noted between 10–40 diffusion encoding directions, while more consistent values were obtained between 40–60 directions.

**Fig 5:**
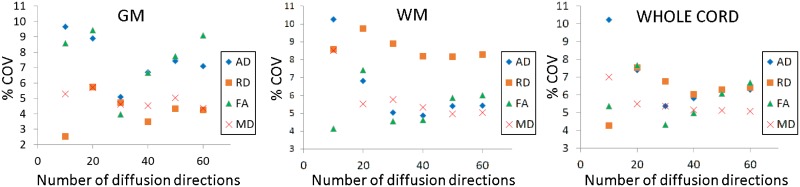
The effect of fitting the DTI model using a different number of diffusion directions on the percentage coefficient of variation (%COV) of the DTI indices, here plotted separately for grey matter (GM), white matter (WM) and the whole cord.

The results from fitting the DTI model with gradually reduced sets of measurements on the %COV of the DTI indices within each tissue-type is demonstrated in Fig 5. This figure shows that, at least in WM and the whole cord, there was a trend towards decreasing %COV values in most of the DTI indices when the model was fitted with 20 or more sets of measurements. In GM, apart from MD and RD, there was no other apparent gain from using a number of diffusion directions higher than 20.

The results of the linear mixed models examining variation in means of the DTI indices within each tissue-type across the number of diffusion directions are shown in Table 3. From the table, two main observations can be made. Firstly, that the MD measures are the least affected in terms of the number of diffusion directions used in the present study. Secondly, in all other DTI indices the variation in mean values becomes less significant as the number of diffusion directions increases, and the percentage size of the largest difference for each measurement also becomes lower.

**Table 3:** Results of the linear mixed models examining variation in means of the DTI indices within each tissue-type between different diffusion encoding directions; significant and non-significant values are shown along with the percentage size of the largest difference for each measurement.

ROI	DTI Indices	Number of diffusion directions					
		10–20	10–30	10–60	20–30	40–60	50–60
GM	AD	P<0.0001 (4.8%)	P<0.0001 (7.2%)	P<0.0001 (8.8%)	P = 0.0565 (2.5%)	P = 0.0056 (1.2%)	P = 0.0873 (0.4%)
	RD	P<0.0001 (10%)	P<0.0001 (12.2%)	P<0.0001 (14.5%)	P = 0.0565 (2.5%)	P = 0.0001 (1.9%)	P = 0.4236 (0.3%)
	MD	P = 0.0055 (2%)	P = 0.0205 (2%)	P = 0.006 (1.9%)	P = 0.33 (0.3%)	P = 0.2626 (0.4%)	P = 0.8342 (0.1%)
	FA	P<0.0001 (15.1%)	P<0.0001 (20.5%)	P<0.0001 (25.7%)	P = 0.0008 (6.4%)	P<0.0001 (4.5%)	P = 0.0017 (1.4%)
WM	AD	P = 0.0004 (3.6%)	P = 0.0019 (4.5%)	P<0.0001 (5.6%)	P = 0.4730 (0.9%)	P = 0.0242 (0.8%)	P = 0.8059 (0.1%)
	RD	P<0.0001 (7.4%)	P<0.0001 (8.9%)	P<0.0001 (10.8%)	P = 0.4131 (1.3%)	P = 0.0219 (1.2%)	P = 0.7854 (0.1%)
	MD	P = 0.5092 (0.5%)	P = 0.7824 (5.1%)	P = 0.951 (0.6%)	P = 0.9965 (0.02%)	P = 0.947 (<0.1%)	P = 0.7128 (0.1%)
	FA	P<0.0001 (6.5%)	P<0.0001 (8.5%)	P<0.0001 (10.5%)	P = 0.1985 (2.2%)	P = 0.0014 (1.5%)	P = 0.6696 (0.1%)
Cord	AD	P<0.0001 (4.1%)	P<0.0001 (5.7%)	P<0.0001 (7%)	P = 0.1525 (1.7%)	P = 0.0042 (0.9%)	P = 0.2843 (0.2%)
	RD	P<0.0001 (8.7%)	P<0.0001 (10.6%)	P<0.0001 (7%)	P = 0.1857 (1.8%)	P = 0.0018 (1.4%)	P = 0.6371 (0.1%)
	MD	P = 0.0884 (1.1%)	P = 0.2510 (1.1%)	P = 0.293 (1.1%)	P = 0.8140 (1.1%)	P = 0.6841 (0.2%)	P = 0.772 (<0.1%)
	FA	P<0.0001 (10.1%)	P<0.0001 (13.9%)	P<0.0001 (17.1%)	P = 0.0133 (4.2%)	P<0.0001 (2.6%)	P = 0.0304 (0.6%)

Abbreviations:- AD = axial diffusivity; RD = radial diffusivity; MD = mean diffusivity; FA = fractional anisotropy; GM = grey matter; WM = white matter.

## Discussion

This study has shown that tissue-specific (i.e. GM and WM) DTI indices within the LSE can be obtained reliably using a commercially available 3T MR system and analysis software. However, in order to achieve the results presented here, several technical considerations had to be addressed such as the choice of the acquisition and readout type, the localisation of the LSE to ensure consistent measurements across all subjects and the reproducibility of measurements.

EPI-based rFOV acquisition variants have recently shown promise for studying the SC [15–21]. The rFOV acquisition and readout scheme used in this work is based on a product sequence [15, 26], which has been shown to be suitable for use at any level of the SC in the axial plane [26]. The sequence offers reduced readout duration, combines fat saturation with outer volume suppression, and enables the reliable acquisition of images of the lower SC, which are free from aliasing and susceptibility artifacts and with sufficiently high resolution to allow tissue-specific assessment. Using this acquisition it was also possible to account for physiological motion through the use of cardiac gating, even though CSF pulsation is known to be considerably lower in the lumbar spine than in the cervical and thoracic spine [38, 39].

Recently, rFOV ss-EPI [26] and large FOV ss-EPI [40, 41] acquisitions have been used to obtain tissue-specific DTI indices in the neurologically intact lower SC. Whilst such studies are necessary to establish reference values in the healthy population in order to better interpret results in neurological disease, it is essential that measurements are obtained consistently, as this enables more meaningful comparisons. For example, positional variation of the lower SC must be accounted for, as has been shown in a recent study suggesting the use of the LSE as anatomical landmark [34]. In the present study, the suggested high-resolution acquisition was included in the protocol, as well as a single b-value diffusion-weighted scan, to identify the LSE and facilitate subsequent inter-subject and intra-subject comparison of quantitative measurements. This allowed normative tissue-specific DTI indices within the LSE to be obtained consistently between-subjects, but also allowed a more robust assessment of the reproducibility of the measurements from repeated scans i.e. within-subject assessment.

The reproducibility of DTI measurements obtained using rFOV ss-EPI acquisitions in the lower SC has not been reported to date although studies using similar acquisitions have provided results in the upper SC [22, 23]. The results from this study have shown that the %COV values were lower than 10% in all cases, when using the original protocol with diffusion encoding applied in 60 directions, and these results are in line with the previous reports in the upper SC. However, results from studies investigating different levels of the SC may not be directly comparable because of various technical reasons such as the b-values used for diffusion encoding or the number of diffusion directions.

In this study, the effect of fitting the DTI model to gradually reduced sets of measurements was examined retrospectively by assessing any changes in the quality (C and CNR measures) and reproducibility (%COV) of the DTI indices. In terms of quality, it was shown that for CNR in particular there was a trend towards increasing values when a higher number of diffusion encoding directions was used, and this effect was more pronounced between 10–40 diffusion encoding directions, while more consistent values were obtained between 40–60 directions. This suggests that there maybe a higher benefit from using 40 or more diffusion encoding directions. In terms of reproducibility, this exploratory study has shown that, even though a surface RF coil was used, the %COV values for all the DTI indices were below 10% when the DTI model was fitted with 20 or more distributed diffusion directions; this result is in agreement with previous general recommendations [42, 43]. Therefore, this exploratory study confirms the feasibility of obtaining DTI indices reliably in the lower SC using a surface coil and quantifies for the first time the effect of the angular resolution of the diffusion encoding protocol in terms of DTI scan-rescan reproducibility.

The results of the linear mixed models examining variation in means of the DTI indices within each tissue-type across the number of diffusion directions have shown that the MD measures are the least affected in terms of the number of diffusion directions used. This is likely due to the fact that MD represents the average diffusivity over the unit sphere, and the mathematical operation of averaging effectively acts as a low pass filter i.e. it is robust towards noise. With regard to all other DTI indices, the variation in mean values becomes less significant as the number of diffusion directions increases, and the percentage size of the largest difference for each measurement also becomes lower, suggesting that there maybe a higher benefit in such measures when more directions are used e.g. 40 or more (Table 3). Of course, the use of a higher number of diffusion directions requires longer examination times, and this may not always be feasible in practice. Nevertheless, the results from this study provide a useful index to guide future applications.

In order to better interpret the DTI indices obtained in this study, one could examine previous measurements obtained in the upper SC using similar methods to establish whether DTI indices vary naturally along the length of the cord; one could also examine previous measurements obtained in the lower SC using similar methods to investigate whether or not the results are comparable. In previous research [23, 24], identical acquisition and analysis protocols as in this work were used to investigate the cervical SC and have obtained GM values for FA (0.56–0.57), MD (0.82–0.92 μm^2^ms^-1^), RD (0.53–0.54 μm^2^ms^-1^), AD (1.41–1.6 μm^2^ms^-1^) and WM values for FA (0.76–0.8), MD (0.91–0.97 μm^2^ms^-1^), RD (0.36–0.39 μm^2^ms^-1^), AD (1.96–2.16 μm^2^ms^-1^). From Table 1 it can be seen that, in comparison, values in the LSE-GM were lower for FA (0.4), AD, (1.2 μm^2^ms^-1^), higher for RD (0.63 μm^2^ms^-1^) and similar for MD (0.82 μm^2^ms^-1^); values in the LSE-WM were lower for FA (0.58), AD, (1.66 μm^2^ms^-1^), higher for RD (0.58 μm^2^ms^-1^) and similar for MD (0.94 μm^2^ms^-1^). These results are in agreement with previous studies showing a reduction in FA and AD in the lower SC (both in GM and WM) as compared to the cervical SC, whereas MD remains relatively consistent throughout the entire length of the cord [26, 41].

Considering the aforementioned methodological differences between this and other rFOV DTI studies of the lower SC, it may not be possible to directly compare the DTI indices obtained here. Two studies have used similar acquisition protocols as in the present study to investigate the lower SC and have obtained WM average values for FA (~0.63–0.67), MD (~0.85–0.92 μm^2^ms^-1^) [26, 27]. From Table 1 it can be observed that, in comparison, values in the LSE-WM in this work were lower for FA (0.58) and higher for MD (0.94 μm^2^ms^-1^). One of the two studies also reported GM values for FA, which were lower than the present study (0.32 and 0.4, respectively) [26]. Possible explanations of these differences could be related to the sample size, the b-values used for diffusion encoding, the number of diffusion directions and the possibility that measurements were obtained from dissimilar regions in the lower SC in each case. Such results emphasise the importance to identify and reduce the sources of errors as much as possible, developing robust imaging protocols to facilitate reliable longitudinal observational studies in neurological disease.

In the present study, variations in the DTI indices at different spinal cord levels were not examined specifically, thus the true magnitude of such potential variations is unknown. However, assuming that the same DTI protocol is employed to study the different levels of the spinal cord each time, hence minimising any influence related to technical factors, any variations in the DTI indices along the length of the spinal cord are likely to be related to microstructural differences within each tissue-type (GM and WM). For example, previous studies in the cervical cord have suggested that there is a higher alignment of white matter fibres at this level, with a proportion of these fibres having a rather large diameter [15, 41, 44]. In turn, this may explain the higher FA and AD (due to the higher alignment of fibres) and the lower RD (as the presence of larger axons reduces the effective diffusion length in both intra and extra-axonal space) values observed in the upper cord as compared to the lower cord, where a progressive decrease in the number of long axons is suspected [26, 41]. Furthermore, previous detailed examination of the cervical cord from C1–C6 demonstrated that higher FA and AD values were mostly obtained at the C2/C3 level and that these values reduced when moving caudally towards the cervical enlargement, possibly due to the higher neural input and output to the upper limbs, which could lead to some disruption of the directional coherence of the nerve fibres on the scale of a voxel [15, 45]. On the other hand, when calculating MD, the influence of the axon/dendrite orientation distribution is factored out, possibly explaining the rather constant MD values along the length of the cord. Differences in DTI indices between the cervical cord and lumbar cord, could be further explained by considering the differences in GM volume fraction at the two levels: GM volume fraction in the cervical cord is 18% whereas in the lumbar cord is 36.3% [46].

In summary, the results of this work demonstrate that it is possible to obtain reliable tissue-specific DTI measurements within the LSE using a clinical MR system at 3T *in vivo*. Future work will aim to translate the acquisition and analysis method presented here to clinical studies of neurological conditions affecting the lower SC.

## Supporting Information

S1 Appendix Variability among individuals in terms of the distance between the lumbosacral enlargement and the surface of the coil. (DOCX)

S1 Dataset Raw data. (XLSX)

S1 Fig The distance between the lumbosacral enlargement and the surface of the coil was determined manually using a line distance tool and verified on both the sagittal (left) and axial planes (right). (TIFF)

S2 Fig Distance between the lumbosacral enlargement and the surface of the RF coil measured in 14 study participants. The plot demonstrates the distribution of the measurements obtained in this study as compared with the median, lower and upper quartile values previously determined from a total of 60 randomly selected scans.
(TIFF)
