# Supplementary material for: Reduced Field-of-View Diffusion-Weighted Imaging of the Lumbosacral Enlargement: A Pilot In Vivo Study of the Healthy Spinal Cord at 3T
Source: PLoS One. 2016 Oct 14;11(10):e0164890. doi: 10.1371/journal.pone.0164890 (PMC5065166; doi:10.1371/journal.pone.0164890)
Supplement: S1 Appendix — (DOCX) [file pone.0164890.s001.docx]

Appendix S1. Variability among individuals in terms of the distance between the lumbosacral enlargement and the surface of the coil.

Variability among individuals in terms of the distance between the lumbosacral enlargement (LSE) and the surface of the coil was studied retrospectively from 60 randomly selected scans, which were previously acquired with identical T2-weighted turbo spin-echo (TSE) and 3D fast field-echo (3D-FFE) sequences. Measurements were taken from the images using a standard line distance tool (S1 Fig.). The median (lower/upper quartile) distance in the 60 scans studied retrospectively was found to be 56.8mm (48.2/62.6mm). S2 Fig. shows the distribution of the measurements obtained from the 14 participants in the present study as compared to the larger number of scans previously investigated.
