# Supplementary figures and images for: Reduced Field-of-View Diffusion-Weighted Imaging of the Lumbosacral Enlargement: A Pilot In Vivo Study of the Healthy Spinal Cord at 3T
Source: PLoS One. 2016 Oct 14;11(10):e0164890. doi: 10.1371/journal.pone.0164890 (PMC5065166; doi:10.1371/journal.pone.0164890)

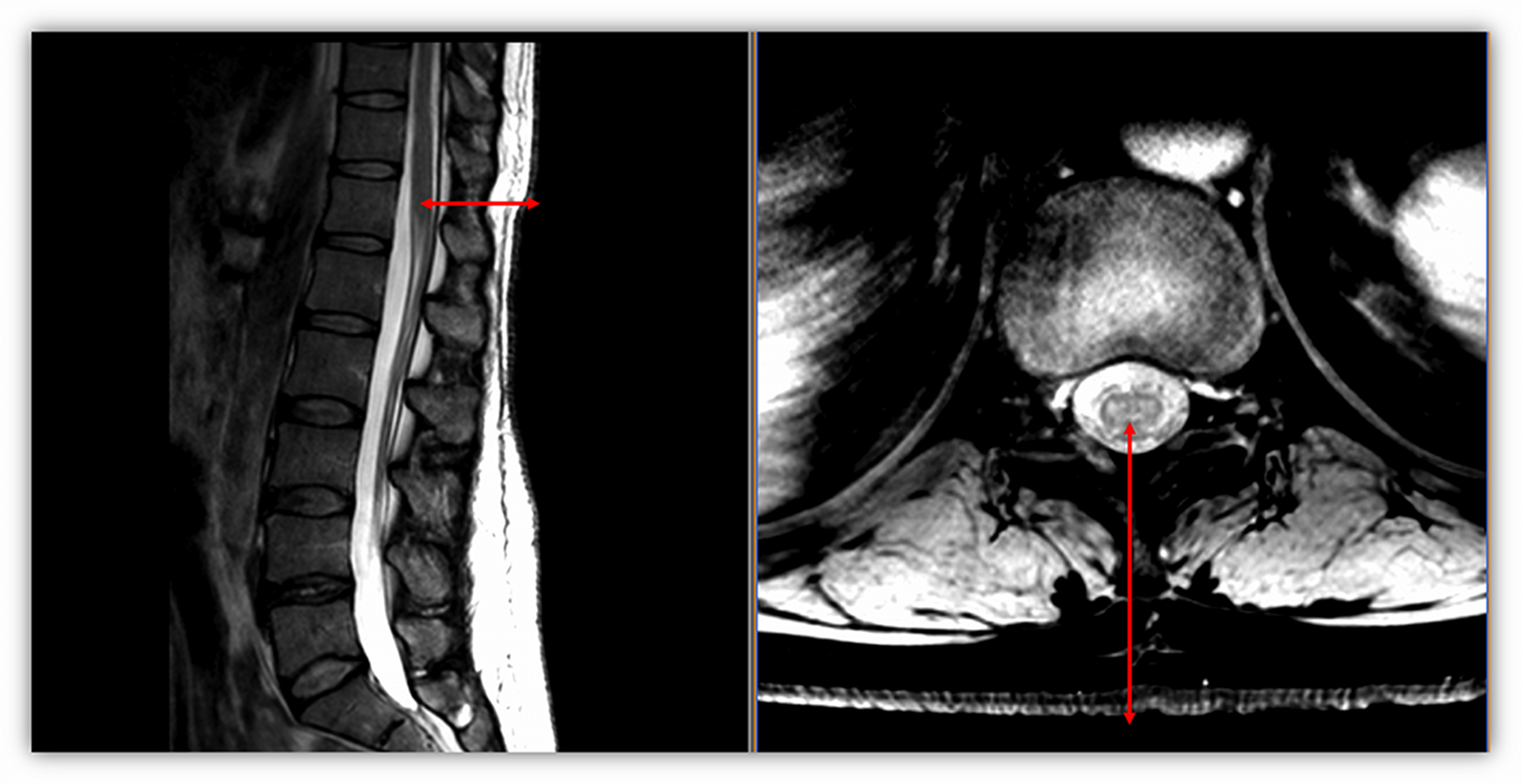

Supplement: S1 Fig — (TIFF) [file pone.0164890.s003.tiff]

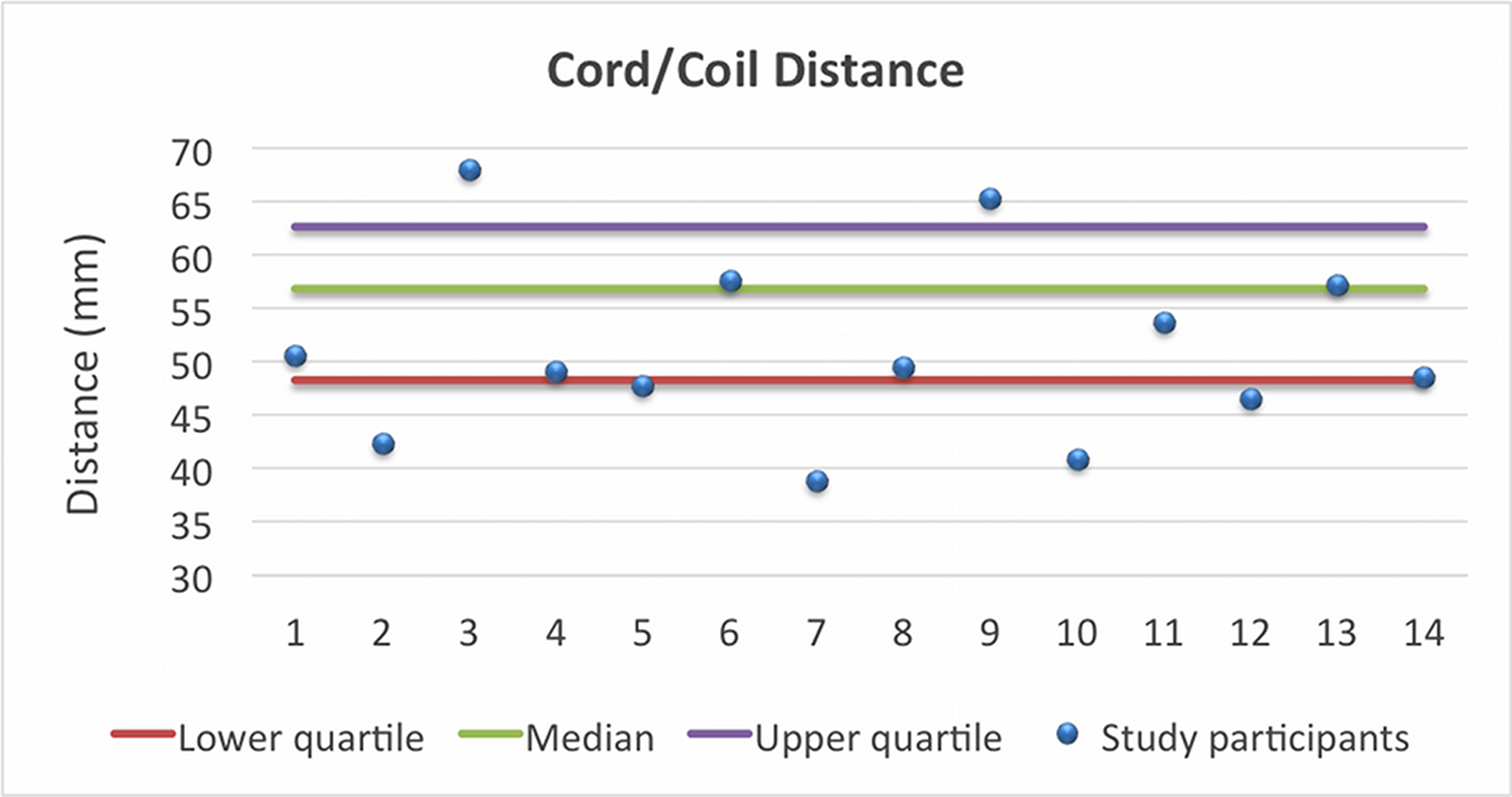

Supplement: S2 Fig — The plot demonstrates the distribution of the measurements obtained in this study as compared with the median, lower and upper quartile values previously determined from a total of 60 randomly selected scans. (TIFF) [file pone.0164890.s004.tiff]
